# Serum biomarkers in oral lichen planus: A biochemical perspective

**DOI:** 10.5937/jomb0-57322

**Published:** 2025-09-05

**Authors:** Jiawei Zhang, Jie Yang, Wei Lu, Shasha Zang, Chengbi Tong

**Affiliations:** 1 Affiliated Hospital of Hebei University, Department of Geratology and Special Hospital Ward, Baoding, China; 2 Affiliated Hospital of Hebei University, Department of Infectious Diseases, Baoding, China; 3 The 82nd Group Military Hospital of the Chinese People's Liberation Army, Department of Laboratory Pathology, Baoding, China; 4 Affiliated Hospital of Hebei University, Department of Laboratory, Baoding, China

**Keywords:** oral lichen planus, angiopoietin-2, vitamin D, IgA, IgG, cortisol, biomarkers, disease severity, immune dysregulation, psychological stress, oralni lihen planus, angiopoetin-2, vitamin D, IgA, IgG, kortizol, biomarkeri, težina bolesti, imuna disregulacija, psihološki stres

## Abstract

**Background:**

Oral lichen planus (OLP) is a chronic inflammatory disease that affects the mucosal tissues of the oral cavity. Its pathogenesis involves immune dysregulation, angiogenesis, and psychological stress. Identifying reliable biomarkers can enhance diagnosis, monitor disease progression, and guide personalised treatment strategies. This study aimed to investigate five key serum biomarkers in OLP patients, including angiopoietin-2 (Ang-2), vitamin D, IgA, IgG, and cortisol, to explore their associations with disease severity.

**Methods:**

This observational, case-control study enrolled 100 OLP patients and 80 healthy controls. The OLP patients were classified into three subtypes: reticular (n=30), atrophic (n=35), and erosive (n=35). Fasting blood samples were collected, and serum levels of Ang-2, vitamin D, IgA, IgG, and cortisol were measured using ELISA, HPLC, immunoturbidimetry, and chemiluminescent immunoassays. Statistical analyses, including t-tests, ANOVA, and Pearson correlation, were performed to assess biomarker levels and their correlations with disease severity.

**Results:**

Ang-2, IgA, and IgG levels were significantly elevated in OLP patients, particularly in the erosive subtype (P<0.001), with positive correlations between these markers and disease severity. Vitamin D and cortisol levels were significantly reduced in OLP patients compared to controls (P<0.01) and showed negative correlations with disease severity. These findings indicate the role of vascular, immune, metabolic, and stress-related factors in OLP pathogenesis.

**Conclusions:**

Ang-2, vitamin D, IgA, IgG, and cortisol are valuable biomarkers for assessing OLP severity and guiding personalised treatment. Monitoring these biomarkers can aid in diagnosing OLP, tracking disease progression, and optimising therapeutic strategies.

## Abstract (sr)

**Uvod:**

Oralni lihen planus (OLP) je hronična zapaljenska bolest koja zahvata mukozna tkiva usne duplje. Njegova patogeneza uključuje imunu disregulaciju, angiogenezu i psihološki stres. Identifikacija pouzdanih biomarkera može poboljšati dijagnozu, omogućiti praćenje toka bolesti i usmeriti personalizovane terapijske strategije. Ova studija je imala za cilj da ispita pet ključnih serumskih biomarkera kod pacijenata sa OLP-om, uključujući angiopoetin-2 (Ang-2), vitamin D, IgA, IgG i kortizol, kako bi se istražile njihove povezanosti sa težinom bolesti.

**Metode:**

Ova opservaciona studija slučaja i kontrole je obuhvatila 100 pacijenata sa OLP-om i 80 zdravih ispitanika. Pacijenti sa OLP-om su klasifikovani u tri podtipa: retikularni (n=30), atrofični (n=35) i erozivni (n=35). Uzorci krvi su uzimani natašte, a serumski nivoi Ang-2, vitamina D, IgA, IgG i kortizola su određivani pomoću ELISA, HPLC, imunoturbidimetrije i hemiluminiscentnih imunotestova. Statističke analize, uključujući t-test, ANOVA i Pirsonovu korelaciju, korišćene su za procenu nivoa biomarkera i njihove povezanosti sa težinom bolesti.

**Rezultati:**

Nivoi Ang-2, IgA i IgG su bili značajno povišeni kod pacijenata sa OLP-om, naročito kod erozivnog podtipa (P<0,001), uz pozitivnu korelaciju između ovih markera i težine bolesti. Nivoi vitamina D i kortizola su bili značajno sniženi kod pacijenata sa OLP-om u poređenju sa kontrolnom grupom (P<0,01) i pokazali su negativnu korelaciju sa težinom bolesti. Ovi rezultati ukazuju na ulogu vaskularnim, imunološkim, metaboličkim i faktora stresa u patogenezi OLP-a.

**Zaključak:**

Ang-2, vitamin D, IgA, IgG i kortizol predstavljaju značajne biomarkere za procenu težine OLP-a i vođenje personalizovane terapije. Praćenje ovih biomarkera može doprineti boljoj dijagnostici, praćenju progresije bolesti i optimizaciji terapijskih strategija.

## Introduction

Oral lichen planus (OLP) is a chronic inflammatory disease that primarily affects the mucosal tissues in the oral cavity [1]. It is characterised by episodes of white, lace-like patterns (reticular OLP) or more severe manifestations, such as erythematous and erosive lesions. OLP is considered an immune-mediated disorder, with T-lymphocyte infiltration playing a central role in triggering inflammation and epithelial damage. Although the exact aetiology of OLP remains elusive, several factors – such as stress, systemic diseases, and autoimmune responses – are implicated in its pathogenesis [2]. OLP affects about 0.1% to 4% of the general population, with a higher prevalence among middle-aged women [3].

The condition poses challenges for both diagnosis and management. Clinically, the reticular form of OLP is typically asymptomatic, while the erosive subtype can cause pain, discomfort, and functional impairments in eating and speaking. In some cases, erosive OLP may increase the risk of malignant transformation into oral squamous cell carcinoma, making regular monitoring essential [4] [5].

OLP's pathophysiology involves a complex interplay between immune responses, angiogenesis, and psychological stress. Immune dysregulation is a hallmark of the disease, with studies showing elevated levels of CD4+ and CD8+ T-cells in affected tissues. Regulatory T cells (Tregs) are also altered, contributing to the chronic inflammatory state [6]. The immune response is further characterised by abnormal production of immunoglobulins, with IgA and IgG levels often elevated in OLP patients, indicating an ongoing systemic immune reaction [7] [8].

Emerging evidence highlights the role of angiogenesis in OLP, particularly in more severe cases. Angiopoietin-2 (Ang-2), a key regulator of vascular remodelling, is involved in inflammatory processes by promoting vascular leakage and immune cell infiltration. Elevated serum Ang-2 levels have been reported in OLP patients, especially those with erosive lesions, suggesting a link between vascular changes and disease severity [9].

Metabolic and psychological factors also play a role in OLP. Vitamin D deficiency, a known modulator of immune function, has been associated with higher disease severity in OLP. Adequate vitamin D levels are essential for maintaining immune tolerance, and its deficiency may exacerbate inflammation in the mucosa [10]. Additionally, chronic stress is known to affect immune function through dysregulation of the hypothalamic-pituitary-adrenal (HPA) axis, resulting in lower cortisol levels in OLP patients. Reduced cortisol may further impair immune regulation, contributing to the persistence of inflammation [11].

Given the multifactorial nature of OLP, a comprehensive approach involving biochemical biomarkers is essential for effective diagnosis, monitoring, and treatment. This study focuses on five key biomarkers-angiopoietin-2, vitamin D, IgA, IgG, and cortisol levels – to better understand their roles in OLP pathogenesis and their potential applications in clinical practice.

## Materials and methods

### Study design and population

This study was designed as an observational, case-control investigation conducted in our hospital over two years, from 2021 to 2023. The aim was to explore the biochemical characteristics of oral lichen planus (OLP) by measuring key biomarkers. The study population was comprised of 100 patients with OLP, confirmed by clinical and histological evaluations. To ensure a representative sample, participants were categorised into three clinical subtypes: reticular (n=30), atrophic (n=35), and erosive (n=35). These subtypes reflect varying levels of disease severity, with reticular lesions often being asymptomatic while the atrophic and erosive forms are more symptomatic and prone to complications.

In addition to the patient group, 80 healthy individuals were enrolled as a control group. The control participants were matched with the patient group based on age and sex to eliminate potential demographic biases. They were selected from individuals attending the same institution for routine health screenings and confirmed to be free from OLP or other immune-related conditions.

### Participant eligibility

Participants were included in the study if they met the following criteria: they had a confirmed clinical and histological diagnosis of OLP, had not used immunosuppressive medications in the six months preceding the study, and did not have systemic conditions known to interfere with immune function. The clinical diagnosis of OLP was made based on the presence of characteristic mucosal patterns, and histopathological confirmation was performed through tissue biopsies.

Exclusion criteria were established to minimise confounding variables. Individuals were excluded if they had concurrent autoimmune or infectious diseases, as these could affect immune and biochemical parameters. Additionally, pregnant and breastfeeding women were excluded due to hormonal influences that might alter biomarker levels and complicate interpretation.

Prior to enrollment, all participants provided written informed consent in accordance with the ethical guidelines established by our hospital.

### Sample collection

Fasting blood samples (4 mL) were collected from each participant between 8:00 and 10:00 AM to minimise diurnal variations. The blood was drawn from the antecubital vein into tubes pre-coated with anticoagulants where necessary. Samples were centrifuged at 3000 rpm for 10 minutes to separate the serum. Aliquots of serum were stored at -80°C until further biochemical analysis.

### Biochemical assays

### Angiopoietin-2 (Ang-2) measurement

Serum levels of Ang-2 were measured using a sandwich enzyme-linked immunosorbent assay (ELISA) kit (R&D Systems, USA). The serum samples and standards were added to wells pre-coated with Ang-2 antibodies. After incubation and washing, a biotin-labelled secondary antibody was applied, followed by streptavidin-horseradish peroxidase. The enzyme-substrate reaction was quantified by measuring the absorbance at 450 nm using a microplate reader, and the Ang-2 concentrations were calculated from a standard curve.

### Vitamin D quantification

Vitamin D levels were determined by quantifying 25-hydroxyvitamin D in serum using high-performance liquid chromatography (HPLC). Briefly, serum proteins were precipitated using acetonitrile, and the supernatant was injected into the HPLC system equipped with a UV detector set at 265 nm. A mobile phase consisting of methanol and water was used to elute the samples. The concentration of 25-hydroxyvitamin D was determined by comparing the sample peak area with that of the calibration standards.

### Immunoglobulin A (IgA) and Immunoglobulin G (IgG) analysis

Serum levels of IgA and IgG were quantified using immunoturbidimetric assays (Roche Diagno stics, Germany). This method involves measuring the turbidity caused by antigen-antibody complexes formed between immunoglobulins in the serum and specific anti-human IgA or IgG antibodies. The turbidity was measured at 340 nm using an automated biochemical analyser. Concentrations were calculated using the calibration curve provided by the manufacturer.

### Cortisol measurement

Serum cortisol levels were determined using a chemiluminescent immunoassay (Siemens Advia Centaur, Germany). In this method, serum samples were mixed with cortisol-binding proteins labelled with chemiluminescent markers. After binding, unbound components were washed away, and a chemical reaction generated light. The emitted light intensity was measured using a photomultiplier tube, with cortisol levels calculated from a standard curve.

### Statistical analysis

All data were analysed using SPSS software (version 26.0). Continuous variables were expressed as mean±standard deviation (SD). The independent ttest was used to compare serum biomarker levels between OLP patients and healthy controls. One-way analysis of variance (ANOVA) was employed to evaluate differences among the OLP subtypes (reticular, atrophic, and erosive). Pearson correlation coefficients were calculated to assess the relationships between biomarker levels and disease severity. For all statistical tests, P-values less than 0.05 were considered statistically significant.

## Results

### Angiopoietin-2 levels and disease severity

Serum levels of angiopoietin-2 (Ang-2) were significantly elevated in OLP patients compared to healthy controls. As shown in Table 1, the mean Ang-2 concentration in OLP patients was 2.98±0.74 ng/mL, whereas the control group exhibited much lower levels, with a mean of 0.52±0.21 ng/mL (P<0.001). Among the OLP subtypes, patients with erosive OLP had the highest levels (3.89±0.78 ng/mL), followed by atrophic (2.42±0.46 ng/mL) and reticular forms (0.76±0.13 ng/mL). Ang-2 levels were positively correlated with disease severity scores (r=0.54, P<0.001), highlighting its potential use as a biomarker for monitoring disease progression and vascular involvement in OLP.

**Table 1:** Etiology and clinical indicators at the admission of patients in different groups.

Group	n	Mean Ang-2<br>(ng/mL)±SD	P-value
Control	80	0.52±0.21	<0.001
Reticular OLP	30	0.76±0.13	<0.001 vs. Control
Atrophic OLP	35	2.42±0.46	<0.001 vs. Reticular
Erosive OLP	35	3.89±0.78	<0.001 vs. Atrophic

### Vitamin D levels and disease severity

Vitamin D deficiency was prevalent in OLP patients, with lower serum 25-hydroxyvitamin D levels compared to controls. As seen in Table 2, the mean level of vitamin D in OLP patients was 19.3±4.5 ng/mL, significantly lower than the 30.5±6.2 ng/mL measured in controls (P<0.01). Erosive and atrophic OLP subtypes demonstrated the most pronounced deficiency, with levels of 16.8±3.9 ng/mL and 18.5±4.2 ng/mL, respectively. A negative correlation was observed between vitamin D levels and disease severity scores (r=-0.48, P<0.01), suggesting that low vitamin D levels exacerbate immune dysregulation in OLP.

**Table 2:** Serum Vitamin D Levels in OLP Patients and Controls.

Group	n	Mean Vitamin D<br>(ng/mL)±SD	P-value
Control	80	30.5±6.2	<0.01
Reticular OLP	30	22.1±4.3	<0.05 vs. Control
Atrophic OLP	35	18.5±4.2	<0.01 vs. Reticular
Erosive OLP	35	16.8±3.9	<0.01 vs. Atrophic

### Elevated immunoglobulin levels

Elevations in IgA and IgG levels were evident among OLP patients. Table 3 presents the mean concentrations of these immunoglobulins. IgA levels were significantly higher in OLP patients (3.4±0.9 g/L) than in controls (1.9±0.5 g/L, P<0.01). Similarly, IgG levels were elevated in OLP patients (12.3±2.1 g/L) compared to controls (9.8±1.8 g/L, P<0.05). The highest immunoglobulin levels were found in the erosive subgroup. Positive correlations between IgA/IgG levels and disease severity (r=0.52 for IgA; r=0.49 for IgG, both P<0.001) suggest that heightened immune responses play a role in OLP pathogenesis.

**Table 3:** Serum IgA and IgG Levels in OLP Patients and Controls.

Group	n	IgA<br>(g/L)±SD	IgG<br>(g/L)±SD	P-value
Control	80	1.9±0.5	9.8±1.8	-
Reticular OLP	30	2.5±0.6	10.7±1.9	<0.05 vs. Control
Atrophic OLP	35	3.1±0.8	12.0±2.0	<0.05 vs. Reticular
Erosive OLP	35	3.9±0.7	13.2±2.3	<0.01 vs. Atrophic

### Cortisol levels and psychological stress

Cortisol levels were significantly reduced in OLP patients compared to controls, as shown in Table 4. The mean cortisol concentration in OLP patients was 8.2±2.1 μg/dL, whereas controls had a mean level of 12.4±3.0 μg/dL (P<0.01). The lowest cortisol levels were found in the erosive group (7.3±1.8 μg/dL), indicating a possible disruption in the hypothalamic-pituitary-adrenal (HPA) axis due to chronic stress. A negative correlation between cortisol levels and disease severity scores (r=-0.46, P<0.01) underscores the role of psychological stress in exacerbating OLP.

**Table 4:** Serum Cortisol Levels in OLP Patients and Controls.

Group	n	Mean cortisol<br>(μg/dL)±SD	P-value
Control	80	12.4±3.0	-
Reticular OLP	30	9.6±2.3	<0.05 vs. Control
Atrophic OLP	35	8.5±2.0	<0.01 vs. Reticular
Erosive OLP	35	7.3±1.8	<0.01 vs. Atrophic

### Correlation analyses between biomarkers

**Figure 1:**
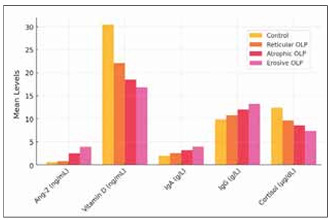
Comparison of biomarker levels across OLP subtypes and controls.<br>This figure presents the mean levels of five key biomarkers angiopoietin-2 (Ang-2), vitamin D, IgA, IgG, and cortisol across different groups: controls, reticular OLP, atrophic OLP, and erosive OLP. The erosive subtype consistently shows the highest levels of Ang-2, IgA, and IgG and the lowest levels of vitamin D and cortisol, indicating a correlation between biomarker levels and disease severity.

The correlation analyses demonstrated significant relationships between the five biomarkers and disease severity. Angiopoietin-2 (Ang-2) and immuno globulin levels (IgA and IgG) were positively correlated with disease severity, indicating that higher levels of these biomarkers reflect increased angiogenesis and immune activation in more severe cases of OLP. In particular, Figure 1 visually illustrates the comparative levels of all biomarkers, Ang-2, vitamin D, IgA, IgG, and cortisol, across different OLP subtypes and controls. The figure highlights the markedly elevated Ang-2 and immunoglobulin levels in the erosive group, reinforcing the role of vascular and immune dysregulation in disease progression.

Conversely, vitamin D and cortisol levels exhibited negative correlations with disease severity, suggesting that lower levels of these biomarkers are associated with more severe clinical manifestations. Spe cifically, the reduction in vitamin D reflects impaired immune modulation, while decreased cortisol indicates the impact of chronic psychological stress on immune function. These findings suggest that both metabolic and psychological factors exacerbate OLP symptoms.

**Figure 2:**
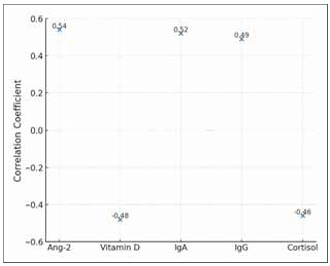
Correlation between biomarkers and disease severity.<br>This scatter plot illustrates the correlation coefficients between the five biomarkers (Ang-2, vitamin D, IgA, IgG, and cortisol) and disease severity in OLP. Positive correlations are observed for Ang-2, IgA, and IgG, suggesting these markers increase with disease progression. In contrast, vitamin D and cortisol exhibit negative correlations, indicating that their lower levels are associated with more severe disease states.

The relationships between these biomarkers emphasise the complex interplay between immune responses, angiogenesis, and psychological stress in OLP pathogenesis, providing potential targets for monitoring and therapeutic intervention. Figure 2 further illustrates the correlation values (r) for each biomarker with disease severity, which shows both positive and negative associations, highlighting the contrasting roles of these biomarkers in disease dynamics.

## Discussion

This study identified significant alterations in the levels of angiopoietin-2 (Ang-2), vitamin D, IgA, IgG, and cortisol in patients with oral lichen planus (OLP), each playing a distinct role in the pathogenesis of the disease. Elevated Ang-2 levels in erosive OLP patients suggest a critical role of angiogenesis in disease progression. Ang-2, a key regulator of endothelial destabilisation, binds to the Tie-2 receptor and disrupts vascular integrity, leading to increased vascular permeability. This process facilitates the extravasation of pro-inflammatory immune cells (e.g., CD4+ T-cells and macrophages) into the submucosa, where they release cytokines such as TNF-α and IL-6, exacerbating epithelial apoptosis and lesion formation. Vitamin D deficiency in OLP patients may impair immune tolerance through its regulatory effects on T-cell differentiation. Vitamin D enhances the activity of T-regulatory (Treg) cells, which suppress autoreactive T-helper 17 (Th17) cells and inhibit the production of pro-inflammatory cytokines like IL-17 and IL-23. In OLP, low vitamin D levels likely disrupt this balance, resulting in unchecked Th17-mediated inflammation and mucosal damage. The elevated IgA and IgG levels observed in erosive OLP indicate systemic B-cell hyperactivity and potential autoantibody production. IgA, a mucosal antibody, may form immune complexes with keratinocyte antigens, triggering complement activation and neutrophil recruitment. Similarly, elevated IgG could target epithelial adhesion molecules (e.g., desmogleins), disrupting cell-cell junctions and promoting erosive lesions. Reduced cortisol levels in erosive OLP patients suggest dysregulation of the hypothalamic-pituitary-adrenal (HPA) axis, potentially driven by chronic psychological stress. Cortisol, a glucocorticoid, normally suppresses inflammation by inhibiting NF- B signalling and cytokine production. In OLP, hypocortisolism may lead to unopposed activation of pro-inflammatory pathways (e.g., IL-6 and IFN-γ), exacerbating mucosal damage.

Ang-2 levels were found to be significantly elevated in OLP patients, especially in the erosive subtype, compared to healthy controls. This result suggests that abnormal angiogenesis and vascular remodelling contribute to the severity of the disease. Ang-2, a known regulator of endothelial function and inflammation, likely facilitates immune cell infiltration, thereby exacerbating mucosal damage in OLP [12]. These findings align with previous studies in other chronic inflammatory conditions that report elevated Ang-2 levels as a marker of disease severity. However, to our knowledge, this study is among the first to demonstrate the specific association of Ang-2 with OLP subtypes and its correlation with disease severity, highlighting the potential role of Ang-2 as a novel biomarker for monitoring OLP progression.

The present study also showed that OLP patients exhibited significantly lower serum levels of vitamin D compared to controls, with the most severe deficiency observed in the erosive subtype. This finding reinforces the growing body of evidence linking vitamin D deficiency to immune dysregulation and chronic inflammation. Vitamin D is known to play a key role in maintaining immune homeostasis, and its deficiency may impair the body’s ability to control inflammation, exacerbating OLP symptoms [13] [14] [15]. While previous research has reported an association between vitamin D deficiency and autoimmune or inflammatory diseases, our study adds to the literature by quantifying the specific relationship between vitamin D levels and OLP severity. These results suggest that vitamin D supplementation may be a beneficial adjunct therapy for OLP patients, particularly those with more severe disease manifestations.

In addition, this study found elevated levels of IgA and IgG in OLP patients, with the highest concentrations in the erosive subtype. The elevation of these immunoglobulins indicates persistent immune activation, which may contribute to sustained mucosal inflammation and tissue damage. Although earlier studies have reported increased levels of immunoglobulins in chronic inflammatory diseases, our findings provide further insight into the specific role of IgA and IgG in OLP pathogenesis [16] [17] [18]. Monitoring these immunoglobulin levels could help track disease progression and provide a valuable marker of immune response to therapy.

Cortisol levels were significantly lower in OLP patients compared to healthy controls, with the lowest levels observed in the erosive subtype. This reduction suggests a disruption in the hypothalamic-pituitaryadrenal (HPA) axis, potentially resulting from chronic psychological stress. Chronic stress exposure is known to dysregulate the HPA axis, leading to a state of “HPA axis burnout” characterised by adrenal exhaustion and blunted cortisol responses. This hypocortisolemic state impairs the body’s ability to suppress inflammation, as cortisol acts as a potent inhibitor of pro-inflammatory cytokines such as IL-6 and IFN-γ. In OLP, diminished cortisol levels may permit unchecked immune activation, facilitating T-cell infiltration and epithelial damage, thereby contributing to the persistence and severity of lesions. Notably, erosive OLP patients, who exhibit the most severe clinical manifestations, demonstrated the lowest cortisol levels, aligning with the hypothesis that stress-induced HPA axis dysfunction exacerbates disease progression. These findings are consistent with psychoneuroimmunological models linking chronic stress to mucosal inflammation and prior research showing that psychological stress influences immune dysregulation in inflammatory diseases [19] [20] [21]. The significant negative correlation between cortisol levels and OLP severity underscores the importance of addressing psychological stress in clinical management. Incorporating stress management strategies, such as cognitive behavioural therapy, could complement conventional treatment by restoring cortisol homeostasis and mitigating immune hyperactivity.

The novelty and strength of this study lie in its comprehensive analysis of five key biomarkers in OLP and the correlations drawn between these biomarkers and disease severity. This study not only confirms findings from previous research regarding immune dysregulation and metabolic changes in OLP but also provides new insights into the role of Ang-2 as a vascular marker in this disease. Moreover, the study’s focus on multiple OLP subtypes allows for a nuanced understanding of how these biomarkers vary with disease presentation, which has not been fully explored in earlier studies.

The identification of Ang-2, vitamin D, IgA, IgG, and cortisol as biomarkers of OLP severity holds significant potential for improving clinical practice. First, serial monitoring of these biomarkers– particularly Ang-2 and immunoglobulins – could serve as a noninvasive tool to track disease progression, especially in high-risk erosive OLP patients. Elevated Ang-2 levels may signal active angiogenesis and vascular leakage, prompting early intervention with anti-angiogenic agents (e.g., bevacizumab) to mitigate lesion formation. Second, vitamin D supplementation could be prioritised for patients with marked deficiencies, as restoring optimal levels may enhance immune tolerance and reduce mucosal inflammation, as demonstrated in pilot studies Saeed et al. [13]. Similarly, cortisol profiling may guide adjunctive stress-reduction therapies (e.g., mindfulness-based interventions) to normalise HPA axis function and dampen immune hyperactivity.

Furthermore, stratifying patients based on their biomarker profiles (e.g., »high Ang-2/ low cortisol« subtypes) could enable personalised therapeutic strategies, aligning with precision medicine paradigms. Future longitudinal studies should validate these biomarkers’ predictive value for malignant transformation, while randomised trials could test targeted interventions (e.g., vitamin D + topical steroids vs. conventional therapy). Integrating biomarker-driven approaches into OLP management may ultimately reduce morbidity and improve patient outcomes.

However, this study also has some limitations. The sample size, particularly when stratified by OLP subtype, was relatively small, which may limit the generalizability of the results. Additionally, the cross-sectional design only provided a snapshot of biomarker levels, preventing the assessment of trends over time or responses to treatment. Furthermore, the study focused on a limited set of biomarkers and did not account for other potential contributing factors, such as genetic predisposition, environmental triggers, or additional metabolic markers. Future research with larger sample sizes, longitudinal designs, and broader biomarker panels is needed to validate these findings and explore new therapeutic targets.

## Conclusion

In conclusion, this study highlights the value of monitoring Ang-2, vitamin D, IgA, IgG, and cortisol levels in OLP patients. These biomarkers can aid in diagnosing the disease, assessing its severity, and guiding personalised treatment strategies. Integrating these biomarkers into clinical practice could improve outcomes for OLP patients by enabling more precise monitoring and targeted interventions.

## Dodatak

### List of abbreviations

OLP, oral lichen planus;<br>Ang-2, angiopoietin-2;<br>IgA, Immunoglobulin A;<br>IgG, Immunoglobulin G;<br>HPA, hypothalamic-pituitary-adrenal;<br>ELISA, enzyme-linked immuno sorbent assay;<br>HPLC, high-performance liquid chromatography;<br>ANOVA, analysis of variance;<br>SD, standard deviation

### Funding

This study was supported by the Baoding Science and Technology Plan Project (Grant no#. 2341ZF140; Title: Clinical study and analysis of vitamin D supplementation to improve the prognosis of elderly patients with H-type hypertension).

### Authors’ contribution

Jiawei Zhang and Jie Yang contributed equally to this work.

### Conflict of interest statement

All the authors declare that they have no conflict of interest in this work.
